# Text mining of online job advertisements to identify direct discrimination during job hunting process: A case study in Indonesia

**DOI:** 10.1371/journal.pone.0233746

**Published:** 2020-06-04

**Authors:** Panggih Kusuma Ningrum, Tatdow Pansombut, Attachai Ueranantasun

**Affiliations:** Department of Mathematics and Computer Science, Faculty of Science and Technology, Prince of Songkla University, Pattani, Thailand; University of Lleida, SPAIN

## Abstract

Discrimination in the workplace is illegal, yet discriminatory practices remain a persistent global problem. To identify discriminatory practices in the workplace, job advertisement analysis was used by previous studies. However, most of those studies adopted content analysis by manually coding the text from a limited number of samples since working with a large scale of job advertisements consisting of unstructured text data is very challenging. Encountering those limitations, the present study involves text mining techniques to identify multiple types of direct discrimination on a large scale of online job advertisements by designing a method called Direct Discrimination Detection (DDD). The DDD is constructed using a combination of N-grams and regular expressions (regex) with the exact match principle of a Boolean retrieval model. A total of 8,969 online job advertisements in English and Bahasa Indonesia, published from May 2005 to December 2017 were collected from bursakerja-jateng.com as the data. The results reveal that the practices of direct discrimination still exist during the job-hunting process including gender, marital status, physical appearances, and religion. The most recurrent type of discrimination which occurs in job advertisements is based on age (66.27%), followed by gender (38.76%), and physical appearances (18.42%). Additionally, female job seekers are found as the most vulnerable party to experience direct discrimination during recruitment. The results exhibit female job seekers face complex jeopardy in particular job positions comparing to their male counterparts. Not only excluded because of their gender, but female job seekers also had to fulfil more requirements for getting an opportunity to apply for the jobs such as being single, still at a young age, complying specific physical appearances and particular religious preferences. This study illustrates the power and potential of optimizing computational methods on a large scale of unstructured text data to analyze phenomena in the social field.

## Introduction

The practice of discrimination in the workplace remains persistent even six decades after anti-discrimination law was introduced. According to the International Labour Organization (ILO) [1], discrimination in the workplace may emerge before hiring at job searching and recruitment process, on the job, or upon leaving the job. Recruitment, a critical gateway to the economic opportunity which determines who can consistently have access to work, is found as one of the stages with high frequent occurrences of discrimination. Therefore, if discriminatory practices occur during recruitment, it can potentially exclude some job seekers even from having opportunities to apply for the jobs. However, this problem may be encountered if discrimination practices can be detected early on during the recruitment process.

Several methods such as mixed methods [2], field experiments [3–6], and vignette studies [7] have been utilized to detect discriminatory practices during the recruitment process. Encel and Studencki [2], for instance, used mixed methods to identify age discrimination toward mature workers in Australia by spreading 700 anonymous questionnaires with the cooperation of three job network providers to clients aged 45 and over. Out of 700 questionnaires, only 163 received responses and the results showed that mature age is a major obstacle in obtaining a job.

As for field experiments, Lee and Khalid [3] investigated racial discrimination in hiring fresh degree graduates in Malaysia. Technically, 3,012 fictitious Malay and Chinese resumes were sent to some companies that published job advertisements, then the callbacks for interview attributable to racial identity were analyzed. The outcomes revealed that race was a more important factor in getting a callback than the quality of the resumes. Bygren, Erlandsson and Gähler [4] performed a field experiment using 2,144 fictitious job applicants in the Swedish labour market and found that there was no evidence of discrimination based on gender and parental status. Besides, Agerström *et al*. [5] carried out a field experiment on 5,636 Swedish and Arab applicants and discovered that Arab applicants received fewer interview invitations than Swedish applicants. Moreover, it was essential for Arab applicants to appear both warm and competent.

In addition to field experiments, vignette studies were also undergone to determine discriminatory practices during the recruitment process. Kübler, Schmid and Stüber [7] supervised this approach to investigate gender discrimination for apprenticeship positions in Germany. In this study, 636 human resource managers were asked to evaluate 3,164 short fictitious CVs. All respondents had to evaluate five vignettes, after that each of them had to evaluate fictitious applicants on the scale of 1 to 10 to determine whether the applicants would reach the next step of the hiring procedure or not. The results showed that women were generally evaluated worse than men on average given other variables were being controlled. Oesch [8] combined a vignette study of 500 fictional CVs with a mass displacement survey on 1,200 workers about two years after losing their jobs to test three explanations of why older job seekers had trouble getting reemployed. The study concluded that two of the explanations which are physical skills and high wages were the factors. Moreover, in both settings, the likelihood of getting an interview reduced as age increased.

Although methods such as mixed methods, field experiments, and vignette studies are good measures to detect and investigate discrimination during recruitment, they rely on researchers to conduct experiments or surveys. Therefore, the design of such an experiment or survey may limit the scope of the study or unintentionally create an environment which greatly differs from the real-life recruitment process. Moreover, researchers have no control over their sample sizes since they depend on the number of responses.

When considering the limitations of the aforementioned methods, job advertisement analysis is an excellent alternative to identify discriminatory practices during the recruitment process. Firstly, job advertisements are natural products of recruitment process so they reflect the true nature of the labour market. Secondly, job advertisements can be good indicators of an organization’s commitment to fair-hiring practices [9] since job advertisements may provide rich information regarding the preferences which employers have towards their targeted applicants. Thirdly, researchers can decide the sample sizes of their studies given already available job advertisements.

Traditionally, job advertisement analysis for identifying discrimination is conducted by content analysis [9–16] or by organizing longitudinal study [17] using printed job advertisements from newspaper or leaflet and banner. Those analyses are supervised by manually coding the contents from printed job advertisements. However, as the technology grows rapidly nowadays, the Internet becomes an important tool for recruitment [18,19] due to its benefits in giving job advertising straightforwardness with no geographic boundaries, increasing the speed of acquisition, shortening the hiring cycle, yet decreasing cost and saving time [20,21]. Some companies are even adopting Artificial Intelligent (AI) to support their recruitment process due to the beneficial use of the algorithms or machine learning techniques to automate decision-making. As a result, online recruitment platforms, online job portals and social networking websites substantially surge [22]. Consequently, the number of job seekers using the Internet for job hunting is continually increasing [23,24].

Sensible with this new trend, several studies recently have employed various updated approaches in identifying discriminatory practices. Among those approaches, online job advertisement analysis is a popular method used by present studies in determining discriminatory practices, especially during the job-hunting process [25–30]. However, most of the studies adopting this approach only used a small number of job advertisement samples and limited their scopes to a particular type of direct discrimination, such as gender [25,28,30], physical appearances [27] or marital status [29]. Besides such limitations, those studies still employed the conventional method of content analysis by manually assigning labels to the text. Such a method becomes ineffective and time-consuming when applied to a large number of online job advertisements available.

Fortunately, this challenge can be overcome by adopting some text mining techniques which allow for the extraction of high-quality information from large-scale unstructured text data [31,32]. Those techniques usually make use of computational and statistical methods to effectively process and analyze text data. Given these advantages, text mining techniques are employed to design a proposed method called Direct Discrimination Detection (DDD) which is used to identify the discriminatory word patterns indicating the occurrences of direct discrimination appear in online job advertisements. Direct discrimination refers to the practices which express explicit and clear distinction, exclusion or preference on one or more grounds which has the effect of nullifying or impairing equality of opportunity or treatment on a certain group of people [1]. The scope of this study encompasses the identification of five types of direct discrimination, they are discrimination based on gender, marital status, age, physical appearances, and religion. The DDD was tested using online job advertisements from the official job portal owned by Subdivision of Manpower Placement and Transmigration Office of Central Java Province Indonesia (bursakerja-jateng.com) which were published from May 2005 to December 2017.

In detail, this paper is organized into four main parts. Firstly, the Materials and Methods part elucidates the designation of the DDD method using N-grams and regular expressions (regex) with the exact match principle of Boolean retrieval model. Secondly, the Results part discusses the findings of this study in three aspects; univariate analysis, bivariate analysis, and three-way cross-tabulation. Thirdly, the Discussion part elaborates on the insights of the underlying causes of the discriminatory practices found from the results of this study. Lastly, Implications on Research and Practice part discusses the potential application of DDD for other studies and highlights the utilization of this study’s outcome for government, employers, job seekers and communities, which is not only to provide an overview about the effectiveness of anti-discrimination laws enforcement in real practice but also to raise the awareness toward anti-discrimination and equality in the society.

## Materials and methods

**Fig 1:**
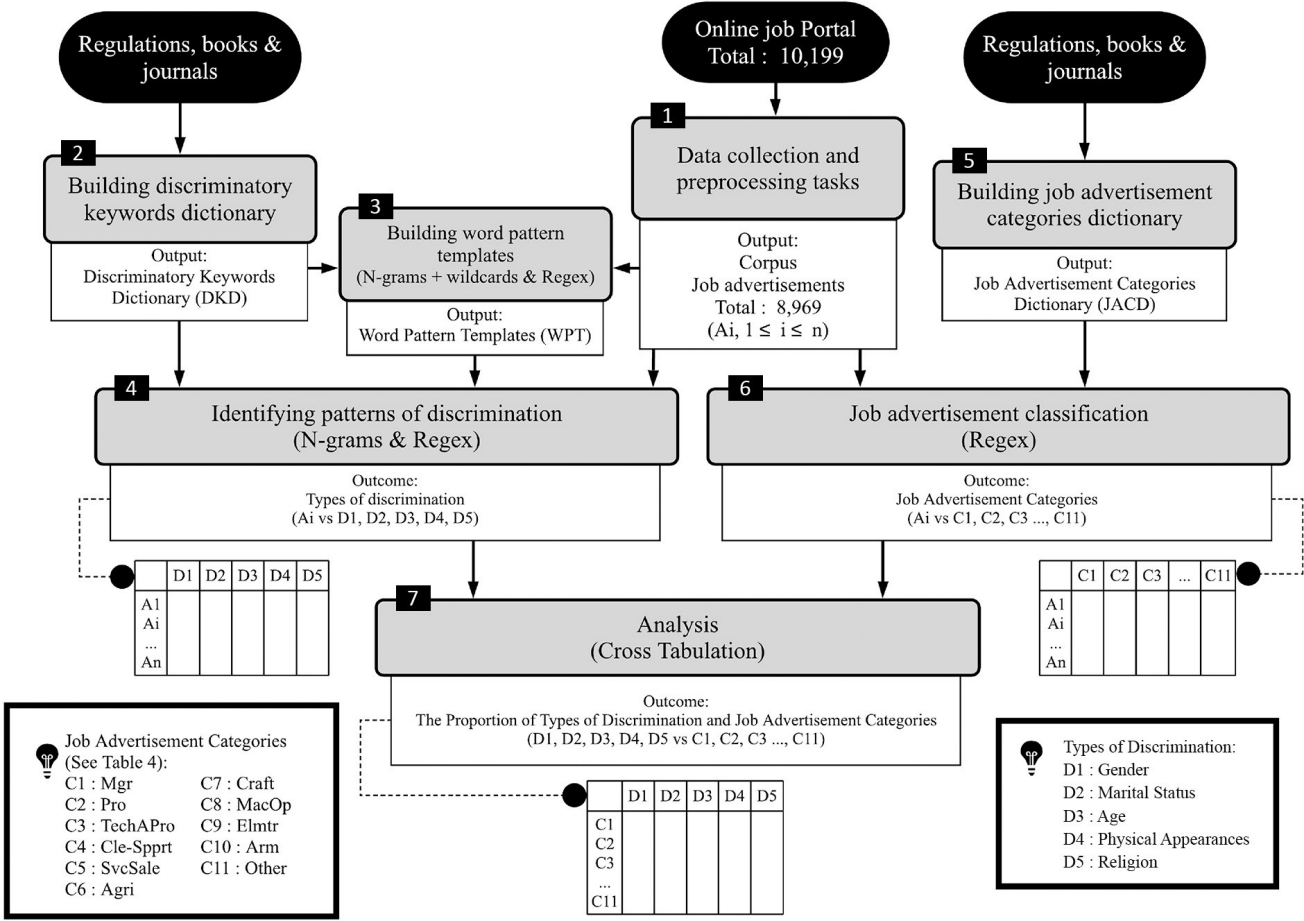
Conceptual framework.

This study proposes a method called Direct Discrimination Detection (DDD) which employs N-grams and regular expressions (regex) with exact match principle of Boolean retrieval model to identify five types of direct discrimination including gender, marital status, age, physical appearances, and religion from online job advertisements. Furthermore, this study uses a quantitative method to accomplish the aims of the research by analyzing and interpreting the statistical results of the outputs from DDD. The conceptual framework of this study is illustrated in Fig 1. There are 5 steps for all the processes to achieve the objectives of this study, namely:

1. Data collection and preprocessing tasks
2. Building discriminatory keywords dictionary
3. Building word pattern templates
4. Identifying patterns of discrimination
5. Building job advertisement categories dictionary
6. Job advertisement classification
7. Analysis

### 1. Data collection and preprocessing tasks

Web scraping is performed to collect the online job advertisements from an official website of the Subdivision of Manpower Placement and Transmigration Office of Central Java Province, Indonesia (bursakerja-jateng.com) which are published during May 2005 to December 2017. A total of 10,199 job advertisements are collected which consist of two languages, namely: Bahasa Indonesia and English.

Every job advertisement consists of:

1^st^ part: a company’s logo or picture
2^nd^ part: the summary which comprises the company name, educational preferences for the position, job position name, company sector, publication date, application deadline, the title of job vacancy, and the capacity of vacant position requested
3^rd^ part: detail information and job requirements (body text)
4^th^ part: a warning note from the website administrator.

Irrelevant hints such as blank page, duplicate advertisements, multiple job vacancies, etc are removed. An initial pool of 8,969 job advertisements is set as the corpus. Later, the job advertisements in the corpus are processed by removing all extraneous materials (e.g., HTML tags, company logos, company names, warning notes, etc). This process leaves the core part of the job advertisements which consists of the 2^nd^ and 3^rd^ part of the job advertisement as the main text data.

Next, data cleansing is performed by checking the misspelling words, eliminating punctuation marks, special characters, tables, columns, and transforming capitalization to lowercase. In this study, the stopwords are not removed from the corpus. Afterwards, tokenization is undergone to transform the raw text from the job advertisements into tokens. In this study, the tokens are formed into words and phrases. Now, all relevant texts in each job advertisement have been tokenized. Hereafter, the tokenized texts in the corpus are ready for further process.

### 2. Building Discriminatory Keywords Dictionary (DKD)

In this study, a knowledge-based approach is chosen to formulate the Discriminatory Keywords Dictionary (DKD). Firstly, books and regulations are reviewed to identify the keywords and terms related to discrimination. The list for sources can be checked in [33–37]. The explanations about each type of discrimination from the sources are used as references in formulating the keywords. Furthermore, the keywords are compiled to construct DKD. Table 1 shows detail keywords in DKD, completed with the explanations from the sources. Synonyms and abbreviations are also taken into account while formulating DKD.

**Table 1:** Discriminatory Keywords Dictionary (DKD).

Type of Discrimination	English	Bahasa Indonesia	Sample Quotes from Sources
Gender	**Female Preferred** <female keywords>		Job advertisements that explicitly state gender for those who can submit applications are a form of direct discrimination. (Panduan Praktis bagi Pengusaha untuk Mempromosikan dan Mencegah Diskriminasi di Tempat Kerja di Indonesia, 2013) Sex can mean either male or female, or a group of people like men or boys, or women or girls. (Equity and Human Rights Commission, 2019)
	Female	perempuan	
	lady(ies)	wanita	
	woman	putri	
	women		
	girl(s)		
	**Male Preferred** <male keywords>		
	Male man men boy(s)	Pria laki-laki putra	
Marital status	**Single Preferred <**single keywords**>**		Marital status is a criterion that is not relevant to job requirements; because work can be done equally well by people who have married or single. Work that requires women to remain single during a certain period so they can travel or be given intensive training is not acceptable. (Panduan Praktis bagi Pengusaha untuk Mempromosikan dan Mencegah Diskriminasi di Tempat Kerja di Indonesia, 2013) Discrimination based on marital status means discrimination against protected groups on the grounds of whether a person is single, married, remarried, divorced, separated, or a surviving spouse and, in employment cases, includes protection against discrimination based on the identity, situation, actions, or beliefs of a spouse or former spouse. (Victorian Equal Opportunity and Human Right Commission)
	Single not married unmarried	Single lajang tidak nikah tdk nikah belum nikah blm nikah belum pernah nikah blm pernah nikah tidak kawin tdk kawin belum kawin blm kawin belum pernah kawin blm pernah kawin tidak menikah tdk menikah belum menikah blm menikah belum pernah menikah blm pernah menikah tidak berkeluarga tdk berkeluarga belum berkeluarga blm berkeluarga belum pernah berkeluarga blm pernah berkeluarga	
	**Married Preferred** <married keywords>		
	Married	Menikah kawin berkeluarga	
Physical Appearances	**Physical distinction <**physical distinction keywords>		Discrimination based on physical appearances means discrimination because of your physical features. Your physical features are your height, weight, size, shape or another bodily characteristic. These also include facial features, hair and birthmarks. (Victorian Equal Opportunity and Human Right Commission)
	Height weight face body physical(ly) good looking good appearance proportional attractive	Tinggi berat badan tubuh wajah penampilan berpenampilan menarik cantikproporsional proposional	
Age	**Age Preferred** <age keywords>		
	Age <digit> year(s)	Usia umur <digit>tahun	Employers should not set age as a condition for work. Word or phrase which impresses the priority of employee candidates from certain age groups also should not use in job advertisements (Panduan Praktis bagi Pengusaha untuk Mempromosikan dan Mencegah Diskriminasi di Tempat Kerja di Indonesia, 2013) Direct age discrimination can be quite wide (for example, by pointing any age group such as ‘people under 50’ or 'under 18s'). It can also be quite specific (for example, ‘people in their mid-40s’). Terms such as ‘young person’ and ‘youthful’ or ‘elderly’ and ‘pensioner’ can also indicate an age group. (Equity and Human Right Commission, 2019)
Religion	**Religious Choices Preferred** <religious choices keywords>		Religion cannot be accepted as a criterion for recruitment except in cases where employees must carry out religious functions as part of job requirements. (Panduan Praktis bagi Pengusaha untuk Mempromosikan dan Mencegah Diskriminasi di Tempat Kerja di Indonesia, 2013) In the Equality Act, religion or belief can mean any religion, for example, Christianity, Judaism, Islam, Buddhism, etc, as long as it has a clear structure and belief system. (Equity and Human Right Commission, 2019)
	Agama beragama islam muslim muslimah non islam non muslim kristenkatolik protestan hindu buddhabudha kong hu cu	Religion moslem non moslem christiancatholic protestant hinduism buddhistbuddhism	
	**Religious Attributes Preferred** <religious choices keywords>		
	Hijab berhijab niqab jilbab berjilbab cadar bercadar islami	Headscarf headscarves	

**Fig 2:**
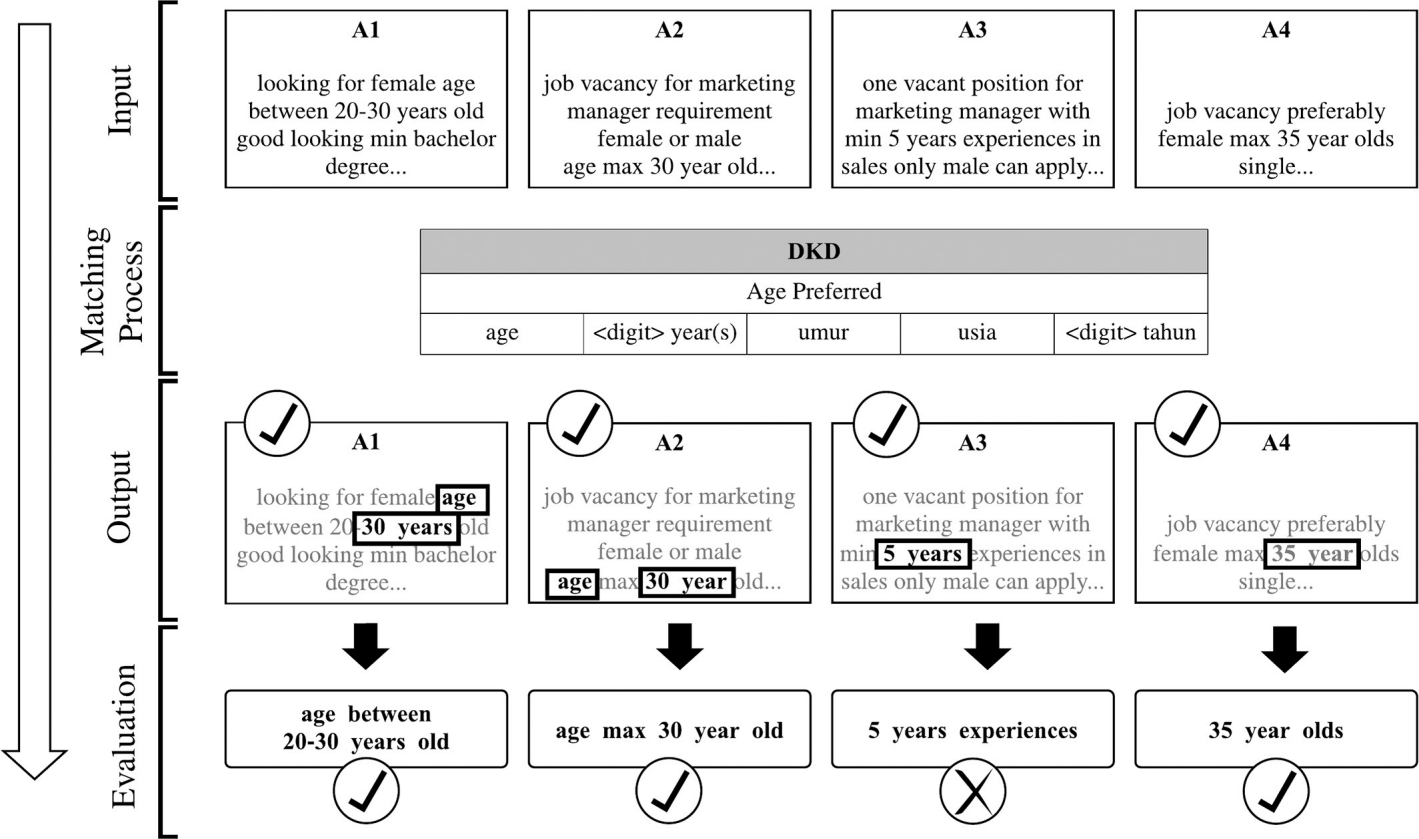
Keyword matching process.

After the DKD has been produced, each keyword will be matched to the tokenized text in each job advertisement in the corpus. Fig 2 illustrates how DDD recognizes the keyword in the corpus and how the keyword matching process is.

As shown in Fig 2, there are four job advertisements namely A1, A2, A3, and A4 as the input. It can be seen that the targeted discrimination keywords are “age”, “<digit> year(s)”, “umur”, “usia”, and “<digit> tahun”. Next, the DDD will match those keywords with tokenized text in all job advertisements. After the matching process, the outputs show that not all the results indicate age preferences. The output from A3 is specifying the duration of job experience. At this point, keywords are not sufficient to identify discriminatory job advertisements as the words order, sequence, and word patterns are crucial. Therefore, building word pattern templates is needed.

### 3. Building Word Pattern Templates (WPT)

Word Pattern Templates (WPT) is a list of templates which shows the combination of keywords and words formed as a phrase or sentence with a specific order. WPT is important to extract more specific information from the text as DKD can only detect the occurrences of keywords but is unable to accommodate the word order, sequence, and patterns.

**Fig 3:**
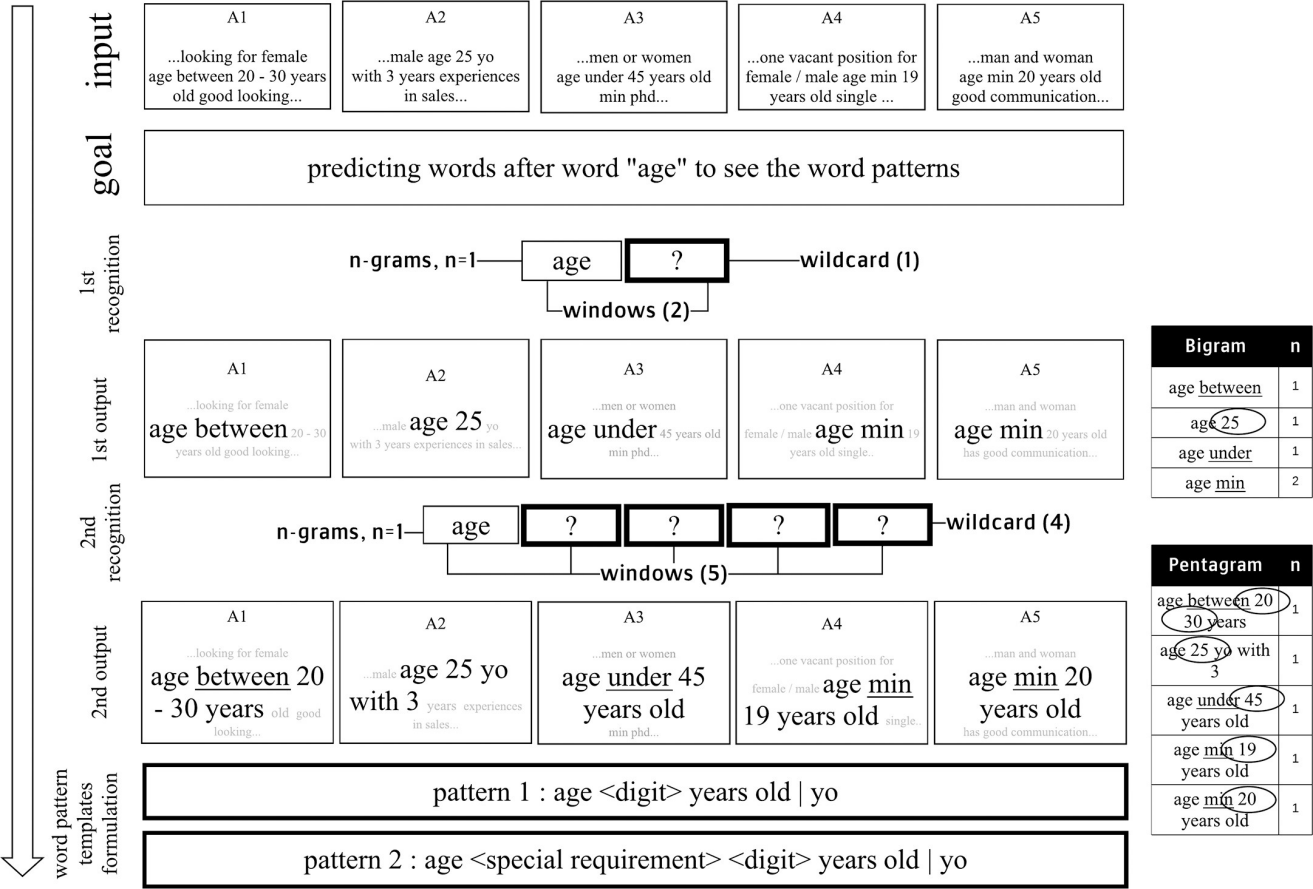
Words pattern recognition and word pattern templates formulation.

To formulate the WPT, DKD and tokenized corpus are used as the inputs during the process in this stage. Furthermore, N-grams operation is utilized to recognize the word patterns from the job advertisements. The maximum N-gram used in this step is 7-grams, while Regex is employed as the complementary to catch the long-distance word patterns with longer than 7-grams in the job advertisements. Finally, Word Pattern Templates (WPT) is constructed as the output. Fig 3 illustrates in detail about WPT recognition process.

As illustrated in Fig 3, four job advertisements namely A1, A2, A3, and A4 are inputted as the data. In this example, the objective is to detect the word patterns that follow the word “age”.

The process is begun by setting the window size and n of n-grams. If the window size is greater than n of the n-grams used, wildcard items will be interspersed among the grounded ones. In this example, the combination of window size two and unigram “age” is set for the 1st recognition. This strategy affects one wildcard item (?) to appear. As shown in Fig 3, the unigram “age” is arranged in the first window. So that, automatically, the second window becomes the wildcard. Hereafter, the wildcard is used to predict the word follow after the unigram “age”.

Afterwards, n-grams will extract words into the wildcard item from texts in each job advertisement. It can be seen from Fig 3 that the operation recognizes some words occurring after the word “age”. They are the words “between”, “25”, “under”, and “min”.

After the first recognition, the window size is extended to detect more detail word patterns from the job advertisement. The list of words obtained from the result of this process furthermore is classified. The word “20”, “30”, “25”, “45”, and “19” are grouped as one variable called <digit>. Meanwhile, the word “between”, “under”, and “min” are compiled as one variable named <special requirement>.

Finally, the patterns templates can be formulated from the outputs of this process. It means the goal to recognize the word patterns that occur after the word “age” is now accomplished. The results can be sum up into two following pattern templates:

Pattern 1: age <digit> years old | yo
Pattern 2: age <special requirement> <digit> years old | yo

However, to optimize the effectiveness of the operation, synonyms and abbreviations were taken into consideration. Adding synonym and extending the query into the WPT have been proven to improve the recall of needed information. Tables 2 and 3 show variables for discriminatory word patterns and the list of WPT. Now, WPT is ready to be used to retrieve information from the tokenized corpus in detail.

**Table 2:** Variables for discriminatory word patterns.

Variables	English	Bahasa Indonesia
**Discriminatory keywords:**		
<female keywords>		
<male keywords>		
<single keywords>		
<married keywords>		
<physical distinction keywords>	(see Table 1)	(see Table 1)
<age keywords>		
<religious choices keywords>		
<religious attributes keywords>		
**Preferences**	Preferably	diutamakan (preferably)
<preferences>	Preferred	utama (prefer)
	Only	khusus (specifically)
**Permit**	Allow	boleh (be permitted)
<permit>	Welcome encouraged	diperbolehkan (be allowed) dipersilahkan (be allowed)
**Specific Requirement**	Min	minimal (minimum)
<specific requirement>	Minimum	maksimal (maximum)
	Max	maks (abbreviate maximum)
	Maximum	mak (abbreviate maximum)
	Valid	belum (not yet)
	Limits	lebih (more than)
	Between	kurang (less than)
	at under	diatas (above)
		melebihi (more than)
		antara (between)
		sampai (to)
		hingga (to)
		s.d (to)

**Table 3:** Word Pattern Templates (WPT) recognized as discrimination.

Type of Discrimination	Pattern Templates
Gender	• male preference
	1. A sentence or phrase with <male keywords> **which does not followed by** <female keywords>
	2. A sentence or phrase with <preferences> followed by <male keywords> **which does not contain** <female keywords>
	3. A sentence or phrase begin with any words **except** <female keywords> followed by <male keywords>
	4. A sentence or phrase with <male keywords> followed by <preferences> **which does not contain** <female keywords>
	5. A sentence or phrase which mentions both <male keywords> and <female keywords> followed by <preferences>
	6. A sentence or phrase which mentions both <male keywords> and <female keywords> followed by <preferences> with <male keywords>
	7. A sentence or phrase which mentions both <male keywords> with <preferences> and <female keywords> followed by <permit>
	• female preference
	1. A sentence or phrase with <female keywords> **which does not followed by** <male keywords>
	2. A sentence or phrase with <preferences> followed by <female keywords> **which does not contain** <male keywords>
	3. A sentence or phrase begin with any words **except** <male keywords> followed by <female keywords>
	4. A sentence or phrase with <female keywords> followed by <preferences> **which does not contain** <male keywords>
	5. A sentence or phrase which mentions both <female keywords> and <male keywords> followed by <preferences>
	6. A sentence or phrase which mentions both <female keywords> and <male keywords> followed by <preferences> with <female keywords>
	7. A sentence or phrase which mentions both <female keywords> with <preferences> and <male keywords> followed by <permit>
Marital status	• single preference
	1. A sentence or phrase with <single keywords> **which does not followed by** <married keywords>
	2. A sentence or phrase with <preferences> followed by <single keywords> **which does not contain** <married keywords>
	3. A sentence or phrase begin with any words **except** <married keywords> followed by <single keywords>
	4. A sentence or phrase with <single keywords> followed by <preferences> **which does not contain** <married keywords>
	5. A sentence or phrase which mention both <single keywords> and <married keywords> followed by <preferences> with <single keywords>
	• married preference
	1. A sentence or phrase with <married keywords> **which does not followed by** <single keywords>
	2. A sentence or phrase with <preferences> followed by <married keywords> **which does not contain** <single keywords>
	3. A sentence or phrase begin with any words **except** <single keywords> followed by <married keywords>
	4. A sentence or phrase with <married keywords> followed by <preferences> **which does not contain** <single keywords>
	5. A sentence or phrase which mention both <single keywords> and <married keywords> followed by <preferences> with <married keywords>
Physical appearances	1. A sentence or phrase with one or more <physical distinction keywords>
	2. A sentence or phrase with one or more <physical distinction keywords> followed by [digit cm] and/or [digit kg]
Age	1. A sentence or phrase with <age keywords> followed by [digit > 10] “year/years” or/and “old”
	2. A sentence or phrase with <specific requirements> followed by <age keywords> [digit > 10] “year/years” or/and “old”
	3. A sentence or phrase with <age keywords> followed by <specific requirements> then [digit > 10]
	4. A sentence or phrase with <age keywords> followed by <specific requirements> then [digit > 10] “years/year” or/and “old”
	5. A sentence or phrase begin with any words **except** “exp/experience/experienced/work/working/bekerja/berpengalaman/pengalaman/berdomisili/bergabung/berlayar/berpengalaman/kontrak/bidangnya/diperpanjang/nomor/no/selama” followed by [digit > 10] “year/years” or/and “old”
Religion	1. A sentence or phrase begin with any words **except** “lecturer/teacher/street/building/university/guru/universitas/dosen/ustadz/ustadzah/sekolah/jalan/jl/gedung/tpa/ilmu/sd/smp/sltp/sma/smu/slta/tk” followed by <religious choices keywords>
	2. A sentence or phrase with <religious choices> followed by <preferences> **which does not contain** “lecturer/teacher/street/building/university/guru/universitas/dosen/ustadz/ustadzah/sekolah/jalan/jl/gedung/tpa/ilmu/sd/smp/sltp/sma/smu/slta/tk”
	3. A sentence or phrase begin with any words **except** “lecturer/teacher/street/building/university/guru/universitas/dosen/ustadz/ustadzah/sekolah/jalan/jl/gedung/tpa/ilmu/sd/smp/sltp/sma/smu/slta/tk” followed by <religious attributes keywords>
	4. A sentence or phrase with <religious attributes keywords> followed by <preferences> **which does not contain** “lecturer/teacher/street/building/university/guru/universitas/dosen/ustadz/ustadzah/sekolah/jalan/jl/gedung/tpa/ilmu/sd/smp/sltp/sma/smu/slta/tk”

### 4. Identifying patterns of discrimination

**Fig 4:**
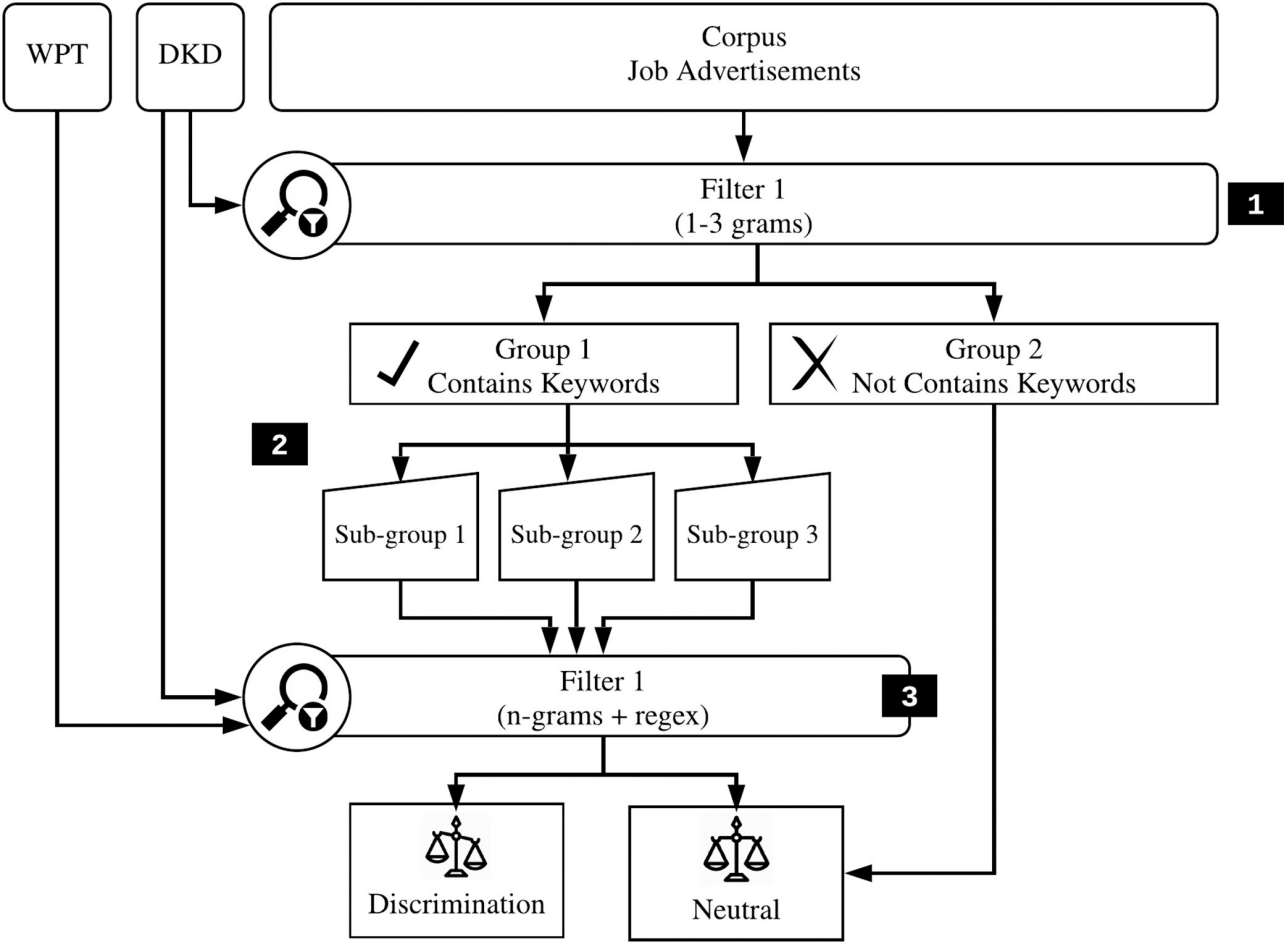
Pipeline for identifying the types of discrimination.

The purpose of this stage is to obtain the identification for each type of discrimination towards job advertisements. To achieve this objective, a list of keywords from DKD is mapped against the corpus using n-grams and regex. However, regex is employed to optimize the system as complementary to catch the long-distance word patterns in job advertisements. Furthermore, Fig 4 illustrates detail about the process for this stage.

Step 1: All the keywords from DKD (Table 1) are mapped from the corpus by using 1 to 3 grams depending on the length of the discriminatory keywords. This step is the first filter for detecting the occurrences of discriminatory words in each job advertisement. This step is found very helpful for giving notification when two or more opposite discriminatory keywords appear in the same job advertisement.
Step 2: All the job advertisements are divided into two groups: 1. Group of job advertisements that contain the discriminatory keywords, 2 Group of job advertisements that do not contain any keywords/terms.
Step 3: Furthermore, the group which contains discriminatory keywords are processed to the next level of n-grams (if the previous step used 1-gram, in this step will be 2-gram and so on). In this stage, the matching process is undergone based on the queries in WPT. This process is the second filter for identifying the pattern discrimination in job advertisements. Finally, the outputs from this process verify whether the keywords are exhibited as discrimination or not.

After finishing the matching process, each job advertisement is labelled based on the pre-chosen queries in WPT. There are three groups for gender discrimination (neutral, male preference and female preference), three groups for marital status discrimination (neutral, single preference and married preference), and three groups for religion discrimination (neutral, religion choice preference and religion attribute preference). While there are two groups for age discrimination (neutral and age discrimination) and two groups for physical appearances (neutral and physical distinction).

### 5. Building Job Advertisement Categories Dictionary (JACD)

As the job advertisement categories have not yet been classified by the website administrator, job advertisement classification is required before further analysis. The ratification of the International Standard Classification of Occupation 2008 (ISCO-08) named Klasifikasi Baku Jabatan Indonesia Tahun 2014 (KJBI 2014) is reviewed as the source for formulating the keywords to detect job advertisement categories. The aim of this process is the Job Advertisement Categories Dictionary (JACD) which consists of keywords list indicated to job categories. To achieve this goal, regex is employed during this process.

According to KJBI 2014 and ISCO-08, there are 10 major groups for job categories in general. However, in this study, it is found that some of the job advertisements use too general terms such as “employee”, “worker”, or “labourer” or use too specific terms for their vacant positions which caused difficulty to recognize which category those job advertisements belong to. To encounter this issue, one additional group called “other” is inserted to accommodate those unclassified job advertisements to the list. Finally, eleven major groups are constructed as described in Table 4. Moreover, S1 Table elaborates more detail about the list of keywords inside JACD where the list involves keywords related to job positions in each job advertisement category.

**Table 4:** Job categories.

Job Category	Label	Description
1	1:Mgr	Managers
2	2:Pro	Professionals
3	3:TechAPro	Technicians and associate professionals
4	4:Cle-Spprt	Clerical support workers
5	5:SvcSale	Service and sales workers
6	6:Agri	Skilled agricultural, forestry and fishery workers
7	7:Craft	Craft and related trades workers
8	8:MacOp	Plant and machine operators, and assemblers
9	9:Elmtr	Elementary occupations
10	10:Arm	Armed forces occupations
11	11:Other	Other

### 6. Job advertisement classification

**Fig 5:**
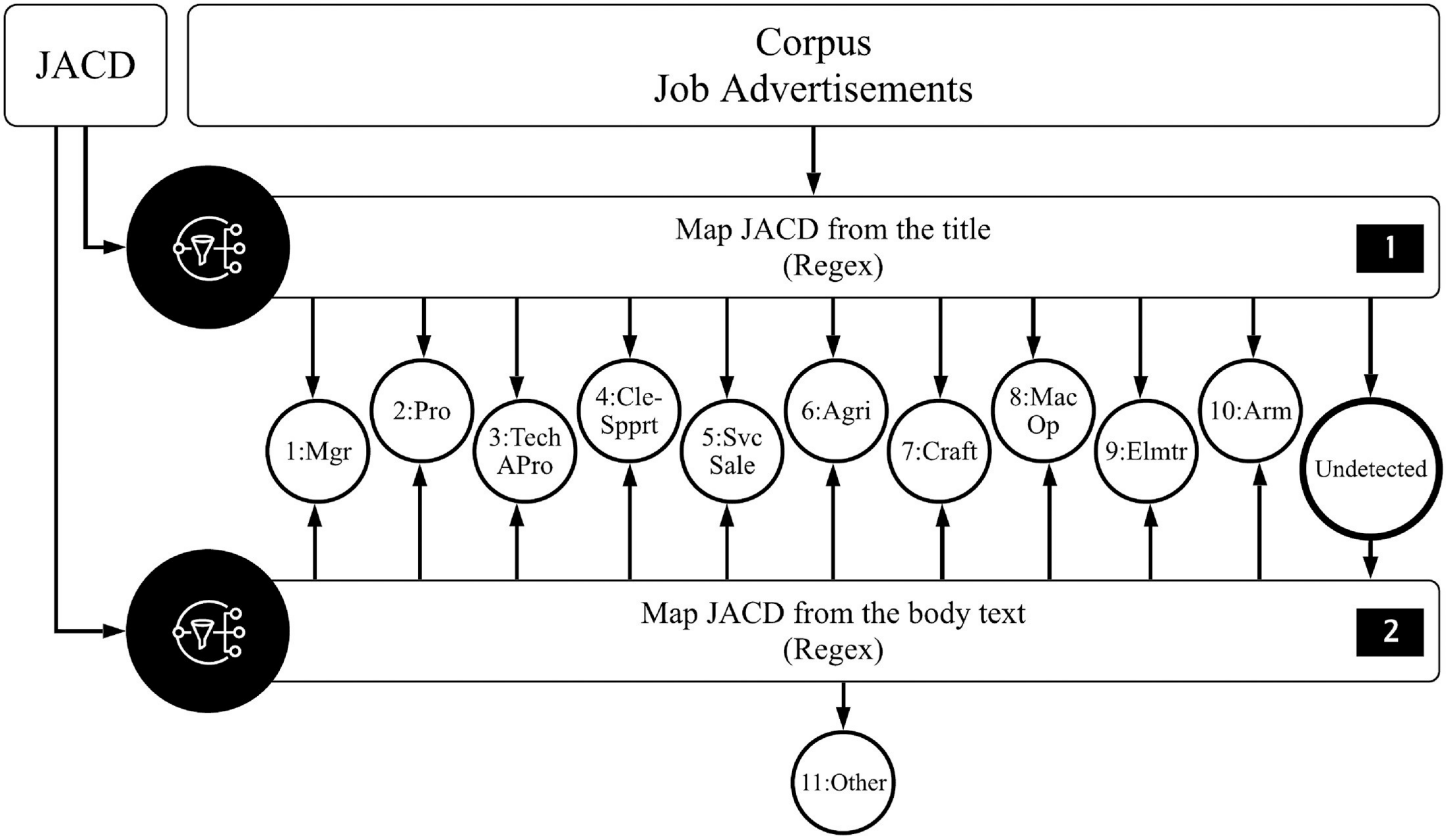
Pipeline for job advertisement classification.

There are several steps involved in this process. JACD and tokenized corpus are utilized as the inputs for this process. Regex is employed to identify the job advertisement categories in the corpus. Fig 5 illustrates the pipeline for this process.

Firstly, regexes map the list of job positions in JACD with the title of the job advertisements. If the title of the job advertisement matches any of the listed job positions, the job advertisement will be labelled according to the job category 1–10 depicted in Table 4.

An issue arose from the fact that some of the job advertisements did not mention clearly about the vacant job position in their titles. Therefore, the second step is employed as additional filter. In this step, the undetected job advertisements from the first operation are mapped again, using its body texts to match the listed keywords in JACD (S1 Table). If the undetected job advertisements match with any of the ten job categories, it will be classified to those job categories. Meanwhile, for those which are still undetected will be labelled as 11: others.

### 7. Analysis

For comparison of the results between the types of discrimination and job advertisement categories, three steps of analysis are conducted including Univariate analysis to explore the distribution and the proportion for all types of discrimination and to identify job advertisement categories, Bivariate analysis to investigate the distribution and proportion of direct discrimination in all job advertisement categories for each type of discrimination and Three-way cross-tabulation to compare the proportions of direct discrimination between two types of discriminations stratified by job categories.

## Results

### 1. Univariate analysis

#### The occurrences of direct discrimination

After all online job advertisements are identified by using n-grams and regular expressions, the results uncover the keywords which indicate the five types of direct discriminations during the job-hunting process. Among the five types of discrimination focused in this study, discrimination based on age (66.27%) and gender (38.76%) are recognized as the most recurrent types of discrimination while the rest are identified in lower frequencies (less than 20%). Table 5 describes the final results of discrimination identification for each type of discrimination.

**Table 5:** Summary of discrimination identification.

Type of Discrimination	N	%
**Gender**		
- Neutral	5,493	61.24%
- Discrimination	3,476	**38.76%**
Male preference	2,154	24.02%
Female preference	1,322	14.74%
**Marital Status**		
- Neutral	8,531	95.12%
- Discrimination	438	**4.88%**
Single preference	412	4.59%
Married preference	26	0.29%
**Age**		
- Neutral	3,025	33.73%
- Discrimination	5,944	**66.27%**
**Physical Appearances**		
- Neutral	7,317	81.58%
- Discrimination	1,652	**18.42%**
**Religion**		
- Neutral	8,873	98.93%
- Discrimination	98	**1.09%**
Religious choice preference	54	0.60%
Religious attribute preference	44	0.49%

In addition to gender discrimination, it is found that employers show their preferences over the male in 24.02% of the job advertisements, while only 14.74% of the job advertisements pinpoint preferences for females. This finding indicates that most employers prefer to hire males rather than females. Besides, in the marital status discrimination type, it can be seen that the number of job vacancies with the requirement for people with unmarried status (439) is notably higher than the number of job vacancies for the people with married status (26). Meanwhile, from the total of 8,969 job advertisements, the results show that up to 1,652 job advertisements express physical appearances discrimination. Nevertheless, there are less than 100 job advertisements that are identified as containing religion discrimination.

#### The distribution of job categories

The results show that job advertisements are highly distributed in the Service and sales workers (24.1%) followed by Technicians and associate professionals (21.5%), Professionals (14.4%), Clerical support workers (12.1%), pPlant and machine operators, and assemblers (8.2%), Managers (6.9%), Craft and related trades workers (6.8%) and less than 10% are distributed in the rest job categories.

Since there are less than 6% of the job advertisements in some job categories, including job category 6 (Skilled agricultural, forestry and fishery workers), job category 9 (Elementary occupations), job category 10 (Armed forces occupations), and job category 11(Others), the job categories are later reorganized into 8 groups. The top 7 groups are kept while those groups which have less than 6% of job advertisements are combined as one group named “Others”.

### 2. Bivariate analysis

Furthermore, a bivariate analysis was undergone. Cross tabulation was utilized to understand the proportion between job advertisement categories and each type of discrimination. The results show that high proportions are found between job advertisement categories and three types of discrimination (gender, age, and physical appearances). Table 6 conveys the detail about the result of each type of discrimination.

**Table 6:** Two-way cross-tabulation between types of discrimination and job categories.

Category	Job Classification	Types of Discrimination															
		Gender				Marital Status				Age		Physical Appearances		Religion			
		0:neutral	1:male	2:female	Total Disc.(%)	0:neutral	1:single	2:married	Total Disc.(%)	0:neutral	1:age pref.	0:neutral	1:physical pref.	0:neutral	1:religious choice pref.	2:religious attribute pref.	Total Disc.(%)
1	Managers	401 (64.57%)	193 (31.08%)	27 (4.35%)	35.43	612 (98.55%)	9 (1.45%)	0 (0.00%)	1.45	273 (43.96%)	348 (56.04%)	570 (91.79%)	51 (8.21%)	618 (99.52%)	3 (0.48%)	0 (0.00%)	0.48
2	Professionals	949 (73.51%)	190 (14.72%)	152 (11.77%)	26.49	1232 (95.43%)	57 (4.42%)	2 (0.15%)	4.57	531 (41.13%)	760 (58.87%)	1167 (90.40%)	124 (9.60%)	1266 (98.06%)	19 (1.47%)	6 (0.46%)	1.93
3	Technicians and associate professionals	1131 (58.78%)	557 (28.95%)	236 (12.27%)	41.22	1843 (95.79%)	76 (3.95%)	5 (0.26%)	4.21	718 (37.32%)	1206 (62.68%)	1761 (91.53%)	163 (8.47%)	1911 (99.32%)	7 (0.36%)	6 (0.31%)	0.67
4	Clerical and support workers	554 (51.06%)	166 (15.30%)	365 (33.64%)	48.94	973 (89.68%)	109 (10.05%)	3 (0.28%)	10.33	275 (25.35%)	810 (74.65%)	772 (71.15%)	313 (28.85%)	1066 (98.25%)	8 (0.74%)	11 (1.01%)	1.75
5	Service and sales workers	1502 (69.41%)	394 (18.21%)	268 (12.38%)	30.59	2065 (95.43%)	92 (4.25%)	7 (0.32%)	4.57	623 (28.79%)	1541 (71.21%)	1490 (68.85%)	674 (31.15%)	2134 (98.61%)	13 (0.60%)	17 (0.79%)	1.39
6	Craft and related trades workers	322 (52.70%)	214 (35.02%)	75 (12.27%)	47.29	595 (97.38%)	14 (2.29%)	2 (0.33%)	2.62	240 (39.28%)	371 (60.72%)	551 (90.18%)	60 (9.82%)	609 (99.67%)	1 (0.16%)	1 (0.16%)	0.32
7	Plants and machine operators and assemblers	305 (41.72%)	285 (38.99%)	141 (19.29%)	58.28	705 (96.44%)	24 (3.28%)	2 (0.27%)	3.55	164 (22.44%)	567 (77.56%)	584 (79.89%)	147 (20.11%)	729 (99.73%)	1 (0.14%)	1 (0.14%)	0.28
8	Others	329 (60.70%)	155 (28.60%)	58 (10.70%)	39.3	506 (93.36%)	31 (5.72%)	5 (0.92%)	6.64	201 (37.08%)	341 (62.92%)	422 (77.86%)	120 (22.14%)	538 (99.26%)	2 (0.37%)	2 (0.37%)	0.74

#### Gender discrimination

More specifically, the results from bivariate analysis reveal that there are more than 30% of job advertisements indicate as having gender discrimination in all job categories except for Professional (26.49%). The highest proportion of gender discrimination is detected from Plants and machine operators and assemblers category. Furthermore, among those discriminatory job advertisements, most of the positions in job categories tend to search male rather than female job seekers. The group of Clerical and support workers is the only job category which shows a predominance of preference toward female compared to male. It can also be seen the highest proportion gap between male and female preferences is found in Managers category, where the preference for males is 31.08% and the preference for females is only 4.35%.

#### Marital status discrimination

The results also display that the occurrences of marital status discrimination are lower than the neutral ones in all job categories. The highest percentage for marital status discrimination is 10.6% in the group of Clerical and support workers, while less than 5% of the job advertisements indicated as having marital status discrimination in the rest of job categories. However, when it happens, among those job advertisements which express marital status discrimination, it is found that the preference for single status predominates the preference for married status in all job categories.

#### Age discrimination

According to the national laws, the minimum working age limit that applies in Indonesia is 15 years old (Act of Republic of Indonesia Number 20 Year 1999 concerning ILO Convention Ratification No.138 concerning Minimum Age for Admission to Employment and Article 1 Act of Republic of Indonesia Number 13 Year 2003 Concerning Manpower) while the maximum age limit for workers to the retirement is 58 years old (Government Regulation of Republic of Indonesia Number 45 Year 2015 concerning the Implementation of the Pension Guarantee Program). The age range for retirement is even wider for government employees which is until 65 years old (Government Regulation of the Republic of Indonesia Number 11 Year 2017 concerning Management of Civil Servants). It can be concluded that the working-age range in Indonesia is between 15 to 65 years old. Therefore, age restriction in job advertisements for any age in this range is unlawful as it will exclude people from having equal opportunity to apply for jobs. Therefore, this study uses these facts as the parameters to set the criteria for age discrimination.

Comparing to other types of discrimination, the results uncover that age discrimination is found as the most frequent type of discrimination in the corpus. There are more than 50% of the job advertisements in all job categories which indicate age discrimination. The highest occurrences for age discrimination are in Plant and machine operators and assemblers group (77.56%). More specifically, it is also found that there are no definite cut points for the age restriction that apply in all job advertisements. The preference for the middle-aged group (45–65 years old) is unpopular for all job categories. Conversely, youngsters are highly requested in all job categories.

**Fig 6:**
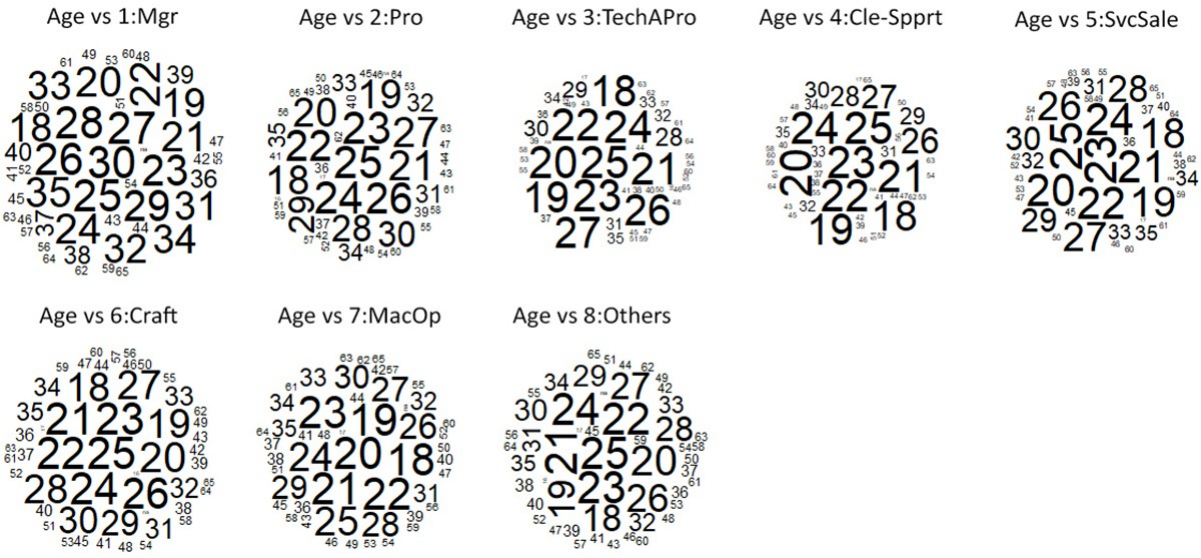
Word cloud age group for all job categories.

It can be seen from Fig 6 that job seekers with the age range of 18 to 35 are highly requested for Managers category. Besides, positions for Professionals, Service and sales workers, Craft and related trades workers, Plant and machine operators and assemblers, and job category Others tend to target job seekers under age 30. Meanwhile, Technicians and associate professionals and Clerical support workers groups give stricter requirements for the age group of job seekers which are under 27.

#### Physical appearances discrimination

Besides, the results convey that discrimination based on physical appearances is found in all job categories. The highest proportion is detected in the Service and sales workers group (31.15%), followed by Clerical support workers (28.85%), Others group (22.14%), Plant and machine operators and assemblers (20.11%), and less than 10% of physical appearances preference is found for job advertisements in the rest job categories.

#### Religion discrimination

Even though the occurrences for religion discrimination are significantly low with no more than 2% of discriminatory job advertisements appear in each job category, these results show that there is still an indication of religion discrimination existence. Additionally, among those job advertisements which indicated as having religion discrimination, it can be seen that both religious choice preference and religious attribute preference are almost equally distributed across all job categories.

### 3. Three-way cross-tabulation

**Fig 7:**
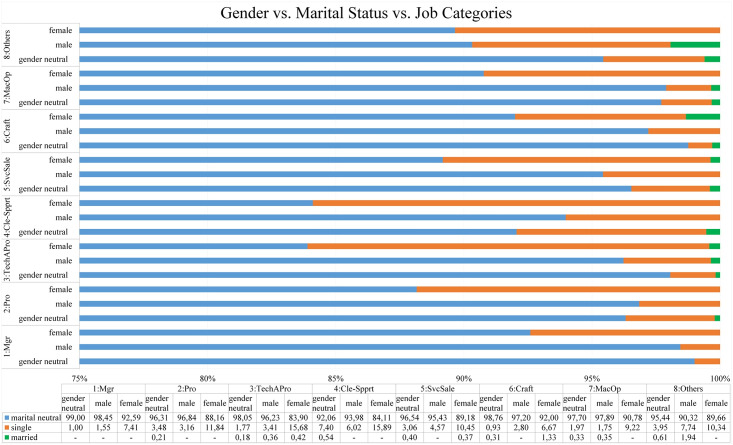
The proportion of three-way cross-tabulation (Gender-Marital Status-Job Categories).

**Fig 8:**
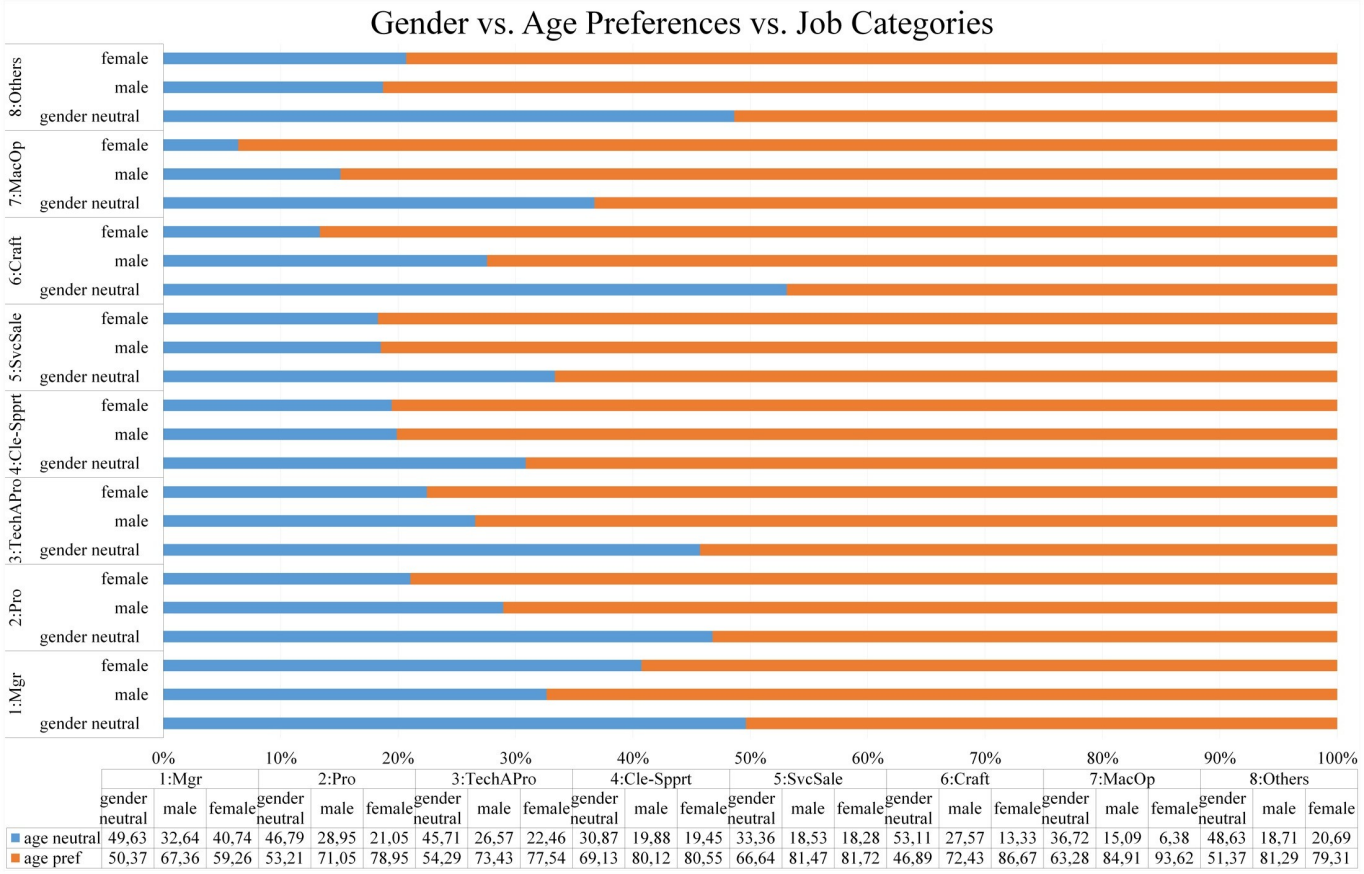
The proportion of three-way cross-tabulation (Gender-Age Preferences-Job Categories).

**Fig 9:**
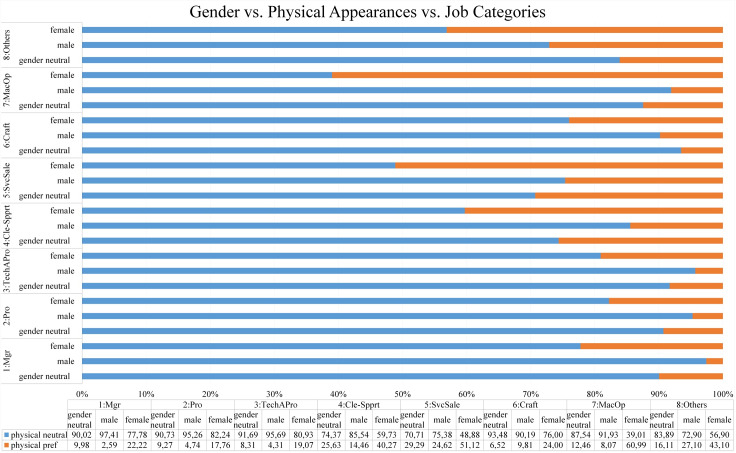
The proportion of three-way cross-tabulation (Gender-Physical Appearances-Job Categories).

**Fig 10:**
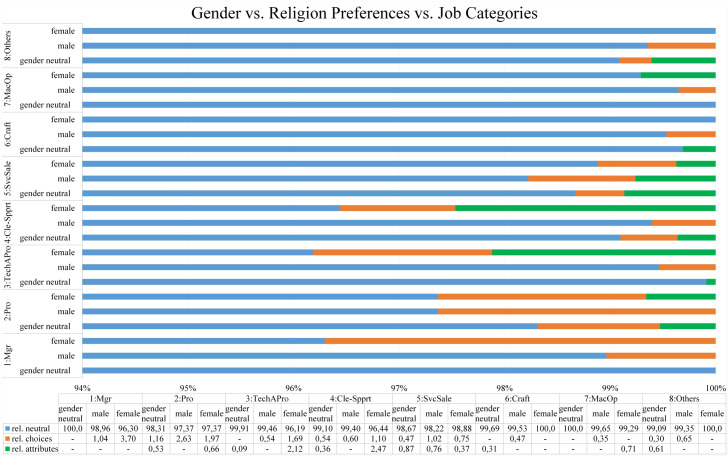
The proportion of three-way cross-tabulation (Gender-Religion Preferences-Job Categories).

In this analysis, three-way cross-tabulation between gender discrimination and other types of discrimination are supervised while every pair of those types is stratified by the job advertisement categories. Furthermore, the proportions from three-way cross-tabulation are calculated in percentage as shown in Figs 7, 8, 9 and 10.

#### Gender vs. marital status vs. job categories

Fig 7 reveals that the preference for job seekers with single status predominates the preference for job seekers who are married in all job categories. Moreover, among the gender preferences group, it can be seen that the proportion of single status is higher for female job seekers compared to male job seekers or gender-neutral in all job categories.

#### Gender vs. age preferences vs. job categories

When it comes to the three-way cross-tabulation between gender discrimination, age preferences, and job categories, the result (as shown in Fig 8) indicates that age discrimination occurs with a high proportion in both male and female preference groups and distributes in all job advertisement categories. Specifically, the proportion for age discrimination in the female preference group is in the range from 59.26% to 93.62%, while the proportion for age discrimination in the male preference group is in the range from 67.36% to 84.91%. In the gender-neutral preference group, the proportion of age discrimination is predominant in all job categories (50.37% - 69.13%) except in the job category for Craft and related trades workers (46.89%).

#### Gender vs. physical appearances vs. job categories

Fig 9 depicts that physical appearances discrimination exists in all gender preference groups and spreads over all job advertisement categories. Moreover, among the gender preference groups, it can be found that the proportion of physical appearances discrimination for job advertisements with female preference is higher compared to male or gender-neutral preferences in all job categories. In detail, the proportion for physical appearances discrimination appears in the range of 17.76% to 60.99% in female preference group while the occurrences of physical appearances discrimination in male preference group and gender-neutral group are in the range of 2.59% to 27.19% and 6.52% to 29.29% respectively. These results show that in most of the job categories, companies prefer to offer job vacancies to male job seekers regardless of their physical appearances while female job seekers have to fulfil some requirements related to physical appearances to get an equal opportunity with their counterparts.

#### Gender and religion preferences vs. job categories

The previous results from univariate and bivariate analysis convey that only a small number of job advertisements indicated as having religion discrimination. The three-way cross-tabulation (as shown in Fig 10) also reveals that there is no significant difference between the proportions of religion discrimination among the gender preference groups in all job categories. However, it can be seen that the proportion of religious discrimination is slightly higher for the female preference group compared to the male or neutral gender group.

## Discussion

Act of the Republic of Indonesia Number 13 Year 2003 Concerning Manpower prohibits discrimination against job seekers in Indonesia. Article 6 of this act states that every job seeker has the same opportunity to get a job without any discrimination. However, the results of this study provide evidence proving that discriminatory practices still exist during job hunting process in Indonesia including gender (38.76%), marital status (4.88%), age (66.27%), physical appearances (18.42%), and religion discrimination (1.09%).

### Ageism

Comparing to the other four types of discrimination focused on this study, the results from univariate analysis (Table 5) convey that age discrimination occurs as the most frequent type of discrimination in job advertisements (66.27%). Moreover, age discrimination happens to every age group (see Fig 6) and there is no definite cut point for the age restriction in the job advertisements. However, middle-aged adults (45–65 years old) tend to be the most vulnerable group which is frequently blocked from all job advertisement categories.

Discussing these phenomena, previous studies had indicated that generally there are some underlying assumptions why age restriction becomes one of the requirements in the job advertisements. According to [38] age restriction by the employer was based on ‘fair innings’ argument. This argument suggested that older workers should make space for younger ones in employment, as they had already had their opportunities to professionally advance in career. Meanwhile, some studies argued that the pervasive reluctance in hiring older people was due to persistent negative stereotypes of older people as hard to train, lack of creativity, overmuch cautious, having difficulty in adopting new technology and lack of flexibility [39]. Following the negative stereotype assumption, Schmidt and Boland [40] highlighted that older worker was considered as eccentrics, curmudgeons (grouchy, angry, uncooperative, nosey/peeping toms), objects of ridicule or the brunt of the joke, unattractive, overly affectionate or sentimental, out of touch with current lifestyle and modern society, overly conservative and afflicted (physically or mentally deficient).

Additionally, the results show that the discriminatory job advertisements which express age preferences occur predominantly across all job categories. Specifically, the findings from bivariate analysis reveal that the occurrences of age discrimination in each job category are in the range of 56.04% to 77.56% while the highest proportion of job advertisements indicating age discrimination appear in plants and machine operators and assemblers job category. Moreover, when comparing the occurrences of age discrimination between genders preference groups in all job categories, the results from three-way cross-tabulation (see Fig 7) convey that there is no tendency toward specific gender regarding the occurrences of age discrimination. Both female and male job seekers considerably experience age discrimination in all job categories.

Align with those findings, McGann et al [41] argued that in manual and traditional working-class occupations such as trade workers, labourers, and machinery operators, older workers perceived ageism as being grounded in negative stereotypes about older bodies. In this kind of job, ageism was associated with the perception that workers with older physique were slower, less fit and more prone to injury.

Moreover, some studies pointed out the fact that age discrimination practice was sometimes seen by employers as a cost-savings technique–circumventing pension payouts and decreasing wages [42–45]. Moreover, health benefit payouts could be held in check, and promotions and on-the-job training opportunities could be reserved for younger workers who were viewed as cheaper and more worthy of long-term investment [46–48].

### Quintuple jeopardy for female job seekers

Besides age discrimination, the findings convey that the occurrences of gender discrimination are also noticeably significant in job advertisements (38.76%). When it comes to the bivariate analysis, there are more than 30% of job advertisements containing gender discrimination in all job categories except for professionals’ positions (26.49%). Moreover, among those job advertisements indicating gender discrimination, the employers’ preferences for male are predominant compared to the preferences toward the female. It can be seen that among eight job categories, the percentage of job advertisements with female preferences is only superior to male in clerical and support workers positions (33.64%).

These findings are in line with previous studies which showed that 7 of the 10 most common jobs for women were sex-segregated such as secretaries, cashiers, nursing aides/orderlies/assistants, servers, etc. [49]. These jobs are characterized by low pay, low status, and short career ladders [50–52].

Furthermore, when gender is linked to marital status, age, physical appearances, and religious preferences, the results from three-way cross-tabulation (Figs 7, 8, 9 and 10) disclose female job seekers tend to face worse discrimination during recruitment process rather than their male counterparts. The results display that the tendency to target female job seekers with single status (on average 10.94%), physical appearances requirements (on average 34.82%), age preferences (on average 79.70%), and religion preferences (on average 1.94%) occurs more persistent in all job categories compared to their male counterparts (on average 3.88%, 11.96%, 76.51%, and 1.01% for single status, physical appearances, age preferences, and religion preferences respectively). Those findings convey female job seekers are preferred to be single, younger, and good looking to get an equal opportunity with their male counterparts to apply for jobs.

Responding to these phenomena, Acker [53] attributed that the very definition of a “job” contained an implicit preference for male workers. Additionally, employers preferred to hire people with few distractions outside of work who could loyally devote themselves to the organization. This preference excludes many women, given the likelihood that they hold primary care responsibilities for family members. Consequently, for many employers, the “ideal worker” is a man [54].

### Complex discrimination in managerial positions

Female job seekers seem to have an immense challenge in managerial job positions while male job seekers do not. Firstly, from the results in bivariate analysis (see Table 6), it appears a wide gap between the proportion of job advertisements with female preferences and the proportion of job advertisements with male preferences in managerial positions. This finding indicates that, regardless of marital status, age, physical appearances, or religion, managerial positions are very demanding for female job seekers to achieve.

Furthermore, when it comes to the three-way cross-tabulation, the results reveal that difficulty is increasing more extremely for female job seekers to work in managerial positions. It can be seen in Figs 7, 9 and 10 that the tendency to target female job seekers with single status (7.41%), physical appearances requirements (22.22%), and religion preferences (3.70%) occur more frequent in all job categories compared to their male counterparts (1.55%, 2.59%, and 1.04% for single status, physical appearances and religion preferences respectively). However, in managerial positions, the results portray that age preference occurs significantly in both female (59.26%) and male preference groups (67.36%) where the highly requested age is found in the range of 18 to 35 (See Fig 6). Those findings depict how female job seekers face massive impediments to be in managerial positions compared to their male counterparts. While male job seekers struggle with the age restriction in applying for this position, female job seekers have to deal with more requirements such as being single, still in young ages, having specific physical appearances, and particular religion preferences.

These phenomena align with previous studies which proved that women were less likely to be hired for high-status jobs [55,56]. It was discussed that the major constraints for women to rise to top managerial positions were gender stereotypes [57]. Leadership itself has been perceived as a masculine concept [58]. Therefore, women are apprehended as incapable of handling professional responsibilities [59], often stereotyped as the less desirable employees, and consequently, not allowed to enter important management positions [60]. Additionally, female executives are stereotyped negatively as behaving aggressive and dominant in their management style. They are also perceived as “bossy” and “pushy” whereas male managers who exhibit such behavior are described as “great leaders” [61].

### Discrimination and challenges for the enforcement of anti-discrimination laws

The overall results of this study describe how apprehensive the phenomena are in the real-life even though a lot of efforts have been made to encounter discrimination. To parse this phenomenon, some potential assumptions from the previous study are discussed to delve into the underlining facts about why law enforcement appears ineffective to tackle the existence of direct discrimination in job advertisements in Indonesia.

The first assumption is due to an underlying presumption toward unconscious discrimination based on implicit bias. The term “implicit bias” is first used by social psychologists Greenwald and Banaji [62]. The implicit bias is developed throughout an individual lifetime and shaped through social influences, culture, and interpersonal relationships or interactions with other social groups [63]. Furthermore, Stypińska [64] explained that in real practice, the employer was often unaware of the fact that some forms of discrimination, such as stating age restriction in a job advertisement is unlawful and can thus be perpetrated.

The second assumption is concerning the classical understandings that have long been trusted for generations by society; they are freedom of association principle and the classic patriarchy culture. It is commonly insisted that freedom of association is one of the fundamental liberties that belong to all individuals in society. That principle is reflected in the rule that most organizations, in this case, employers, can decide whom they want to invite as members and on whom they want to exclude [65]. Meanwhile, the deeply rooted patriarchal culture has been shaping the Indonesian society in responding and thinking about discrimination, especially related to gender discrimination. For the society which is influenced by patriarchy culture, it becomes natural that men are greatly supported by a set of social structures to be dominant over women [66]. Besides, men are perceived to assume the major responsibilities in their families and communities. This strong classic patriarchy notion furthermore becomes a catalyst for gender equality.

The third assumption is related to the problem during the supervision of the industrial activity. The fact indicates that it is very challenging for Indonesia to encounter the discrimination practices as the inspector staff who are responsible for the supervision of labour law in the industrial practices are deficient. The Ministry’s Labor Supervision and Health and Work Safety Director General of the Republic of Indonesia Maruli A. Hasoloan mentioned that in 2017 Indonesia nationally still needed 2,676 staff for inspector positions [67].

The last assumption is regarding the compliance failures to the anti-discrimination laws by society. This condition may cause the enforcement of anti-discrimination laws to be inefficient. Also, it may lead to a fiasco in presenting a sense of deterrent over the penalty and sanction for the anti-discrimination offender. According to the Organization for Economic Co-operation and Development (OECD), there are some factors which contribute to compliance failures [68]. They are related to regulatory knowledge by society, the willingness of the society to comply with the rules, and the ability of the society to comply with the rules. These factors should be taken into account by the policymaker to tackle the compliance failures toward anti-discrimination laws.

### Implications on research and practice

Along with the rapid development in technology, the options for data sources, the methods for data collection, and the latest types of data are growing and expanding. This study takes advantages of the new data availability and breakthrough technological development to demonstrate the power and potential of employing computational methods to analyze a large scale of unstructured text data from online sources to realize and examine phenomena in the social field.

Direct Discrimination Detection (DDD) method, purposed in this study, utilizes text mining techniques to identify how word patterns which indicate direct discrimination are formed in job advertisements. The results reveal the occurrences of discriminatory job advertisements in various job categories and give depiction about the association between each type of discrimination with each job advertisement category in Indonesia. This method is opened for possible improvement in the future by allowing the addition of new discriminatory keywords, the expansion and construction of word patterns, and the extension of query patterns. As a result, the DDD can expectedly be applicable in other job advertisement corpora, for identifying other types of discrimination, and for classifying different job advertisement categories.

In terms of social implication, the findings of this study can be beneficial to the government who is the policymaker as well as employers and job seekers who are stakeholders.

Firstly, the findings of this study are essential to the owner of the dataset used in this study, Subdivision of Manpower Placement and Transmigration Office of Central Java Province, Indonesia. The outcomes discovered in this study can assist the office in reevaluating their current policy of what is considered as lawful job advertisements published on their website. They can furthermore be used in developing strategies for protecting job seekers from discriminatory practices during the job-hunting process.

Secondly, any government and non-government agencies can adopt the DDD method to automatically detect discriminatory online job advertisements and give recommendation before they will be published. Therefore, one of the polices recommended to the government can be requiring that all job advertisements being published pass some test provided by the DDD method. This implementation may help the problem related to the lack of inspector staff in the supervision of labour as the system once implemented can be maintained with less manpower.

Thirdly, if a system that employs DDD method is widely used in practice in Indonesia, the government can collect the outcomes of data analysis to make laws and policies that focus on preventing the types of discrimination that occur often. These laws and policies need not only impose punishments for those who violate but also give rewards for those who cooperate, such as benefits from cooperate tax cut. Additionally, if the government can identify the groups of people who are venerable to be discriminated against, it can introduce some policies to increase their desirability in the job market such as low-cost specific-skill job training. This solution may not directly lessen the occurrences of discrimination during the hiring process, but it can certainly provide job opportunity for those who are victims of discrimination.

The results of this study reveal outcomes that can be utilized by employers, job seekers, and communities as guidance. In more detail, employers can use discriminatory keywords and patterns found in this study as references to guide them in assembling ethical job advertisements. Besides, job seekers and communities can examine the findings to gain more understanding about their rights and the laws that protected them. As a consequence, the results of this study should generate public awareness about the existence and frequency of discriminatory practices that occur during recruitment as well as the apprehension of how anti-discrimination laws can be applied to protect job seekers.

However, this technological approach of detecting direct discrimination in online job advertisements using DDD method is only the beginning step into preventing discrimination during the recruitment process. Although there is no job advertisement containing discriminatory texts, it is still possible that discriminatory practices can occur later on during the hiring process. As long as people have implicit bias, mistakenly believe in freedom of association, and live in patriarchy culture, some organizations will intentionally or unintentionally fail in compliance with anti-discrimination laws. After all, awareness and knowledge are two important key concepts. If we have a system that can detect and explicitly show directed discriminatory texts in job advertisements, job seekers will become aware of anti-discrimination laws and employers will gain knowledge about what exhibits discriminatory practices. In turn, we hopefully can move the practices of anti-discrimination into later stages of recruitments as job seekers require that employers treat them lawfully.

## Conclusions

This study reveals that direct discriminations in job advertisements in Indonesia do exist and spread over many job categories. To achieve the objective of this study, up to 8,969 online job advertisements are analyzed in this study using text mining techniques. A method called Direct Discrimination Detection (DDD) is proposed using the combination of n-grams and regular expressions with a Boolean retrieval model. This method does not only detect the word pattern related to direct discrimination, but it also provides the occurrences of each type of discrimination, the association of each type of discrimination to job categories, also the proportions from three-way cross-tabulation among them. The results convey that age discrimination occurs as the most frequent type of discrimination in the job advertisement (66.27%), followed by gender discrimination (38.76%), physical appearances (18.42%), marital status (4.88%), and religion discrimination (1.09%). Finally, the findings reveal that female job seekers tend to face worse discrimination during the job-hunting process rather than their male counterparts. Moreover, in specific job positions, female job seekers have to face complex jeopardy, not only for being excluded due to their gender, but they also have to fulfil more requirements including being single, still in young age, complying specific physical appearances and particular religious preference, only for having an opportunity to apply for the jobs.

## Supporting information

S1 Table Job Advertisement Categories Disctionary (JACD). (PDF)
