# Supplementary material for: Text mining of online job advertisements to identify direct discrimination during job hunting process: A case study in Indonesia
Source: PLoS One. 2020 Jun 4;15(6):e0233746. doi: 10.1371/journal.pone.0233746 (PMC7272088; doi:10.1371/journal.pone.0233746)
Supplement: S1 Table — (PDF) [file pone.0233746.s001.pdf]

Job Advertisements Categories Dictionary (JACD)

1. Manager	2. Professionals	3. Technicians and associate professionals	4. Clerical support workers	5. Service and sales workers	6. Skilled agricultural, forestry and fishery workers	7. Craft and related trades workers	8. Plant and machine operators, and assemblers	9. Elementary occupations	10. Armed forces occupations	11. Other
manager	ahli	asisten*	admin	sales	petani	produksi	operator	ob	taruna	magang*
manajer	pakar	ass*	adm	salesman	pertanian	production	opeartor	og	taruni	freelance*
kepala	expert	assisten*	data entry	telesales	peternak	pekerja	mekanik	housemaid	polisi	freelancer*
kabag	professional	executive assistant	adminitrasi	salesgirl	nelayan	pengecatan	mechanic	plrt	tentara	free lancer*
hrm	professionals	executive assisstant	entry data	salesperson	pemburu	pembelah	driver	prt	tni	part time*
mpr	dokter	field assistant	input data	agen	penangkap	pemotong	tabber	pembersih	polri	permagangan*
dpr	medis	lab tech	mengetik	agency	agronomi	piping	perakit	buruh	tamtama	
dpd	dentist	ass manager	administrasi	steward	butchee	pemecah	bogger	packer	bintara	
dprd	bidan	ga	aministrasi	waitre	butcher	pemahat	mechanical	cheker		
pejabat	paramedis	assistent*	sekretaris	waiters	penyadap karet	tukang	dragline	pemetik		
direktur	psikolog	assistent manager	sekretarias	waitres	penghasil susu	pembuat	perakit	penyortir		
chief	psikiater	leader	sekertaris	pramugari	pembiak anjing	perancah	awak	porter		
cfo	guru	pelaksana	secretary	waitress	pengumpul subsisten	pemasang	masinis	kuli		
ceo	pendidik	canvasser	pembukuan	barista	tukang kebun	pengecat	pengemudi	pesuruh		
rektor	teacher	canvaser	panitera	marketing	tukang taman	setter	sopir	pengantar		
dekan	dosen	teknik	transkripsionis	cmo	supervisor pertanian	pembersih	supir	delivery		
dean	lecturer	tehnisi	stenografer	representatif		printing	boatswain	pemulung		
rector	pengajar	teknisi	kasir	representative		patterner	pekerja tambang	stuffing person		
director	artist	kanvaser	cashiers	pemasaran		pelebur	juru mudi	pengepul		
head	artis	ppic	cashier	penjualan		pertenunan	juru minyak	kurir		
legislator	animator	technical	teller	pramusaji		carpenter	operator produksi	serabutan		
principal	geologist	teknisi	staf field	waitress		penyambung	production operator	helper		
governor	ilustrator	technician	inventori	security		pandai	staff treatment tangki	marker		
pemimpin	illustrator	dekorator	inventory	satpam		pemoles	roof botler	bellboy		
president	seniman	herbalist	kolektor	guard		pengasah	opt mesin	kernet		
project builder	perawat	dukun	penagihan	pengamanan		penajam	operator mesin	juru bantu		
chief executive	nurse	staff	colector	lifeguard		penyetel	mechanic staff	general worker		
promotion executive	accounting	staf	collector	satpam		penyetem	pekerja penggalian	tenaga bangunan		
vice president	accouting	tekhnisi	bandar	shopkeeper		penggosok	tenaga grader	asisten operator mesin		
ka bag	accountant	elektromedis	rentenir	brandcomm		pengukir	machinery spindle	tenaga perkebunan		
head of	arsitek	elektromedik	front desk	massanger		penjahit	pekerja pengeboran	cleaning service		
ka\sbag	arsitektur	biomedika	inkaso	markeeting		pengetsa	kepala kelasi	domestic helper		
ka bag kantor	it	medical record	kolektor	capster		artwork	anak buah	oyster shucker		
	ti	koordinator	collector	instruktur		garment	juru pemroses	tenaga packing		
	drone specilaist	superintendent	desk colection	marketer		sourching	juru Vulkanisir	tenaga kebersihan		

1. Manager	2. Professionals	3. Technicians and associate professionals	4. Clerical support workers	5. Service and sales workers	6. Skilled agricultural, forestry and fishery workers	7. Craft and related trades workers	8. Plant and machine operators, and assemblers	9. Elementary occupations	10. Armed forces occupations	11. Other
	hrd	coordinator	desk collection	broker		procurement	operator mesin	office boy		
	radiolog	radiografer	executor	pialang		pelapis	juri api	office girl		
	apoteker	radigrapher	eksekutor	penaksir emas		pelukis	cutting handernife	pekerja sampah		
	farmakolog	radiographer	aro	hairstylist		pemintalan	makanik utility	pemetik buah		
	botanist	korlap	mantri	beauty therapis		penjilid		pedagang asongan		
	zoologist	hse staff	checker	beauty advisor		montir		tukang parkir		
	chemist	staf hse	ticketing staff administration	beautician		pembangun		asisten rumah		
	statician	staff keuangan	executive	beautycian		pemasang		pantry		
	fisioterapis	staf operasional	ticketing officer	terapis		penyembelih		plrt		
	acounting	ie staff	penagih	therapis spa		riggers		p l t		
	biologist	chiropractor	resepsionis	spa therapist		perkayuan		pembantu rumah		
	bagian keuangan	buyer	reseptionis	spa theraphis		pertukangan		tenaga cuci		
	industrial engineering	purchasing	recepcionist	therapist		product technician		staff lectra pola		
	penasihat	osteopath	receptionis	phlebotomist		pengawet		juru bersih		
	advisor	sanitarian	reception	housekeepers		pengekstrak		tenaga setrika		
	adviser	panitera	receptionist	housekeeping		cutter		loper koran		
	scientist	ekspor	reseptionist	housekeeper		cutting		pelinting rokok		
	konsultan	impor	recepsionist	butler		finishing		pengantar koran		
	consultant	exim	frontliner	waiter		finisihing		penyemir sepatu		
	counsultant	export	frontliners	penjaga		finshing		office boy		
	researcher	import	front liner	pengawal		penilai		general workers		
	research	pengawas	front line	watchman		pengolah		general worker		
	tentor	estimator	recepsionis	watchwoman		penyetel		clnng service		
	auditor	asm	cs	spg		penyulam		tenaga spon		
	mikrobiolog	drafter	customer relations	spb		instalation		office girl		
	trainner	draftsman furniture	cso	telemarketing		penguji		doorman		
	audit	fotografer	c s o	petugas logistik		penyamak		bongkar muat		
	planner	fotografer	organizer	entrance gate		pembasmi		staff pola		
	surveyor	draffter	compliance	field officer		staff teknik		cleaning service		
	survey	draftman	pengantar surat office	promotion		sewing		clnng service		
	sruveyor	cook	management	lending officer		spray		tukang semir		
	desainer	foreman	license officer	promosi		electricians		tenaga bangunan		
	trainer	forelady	liceuse officer	counter		electrician		tukang sampah		
	recruiter	ie staff	reservation clerk	pramuniaga		electrik		tukang sapu		
	recruitment	industrial engineering staff	bagian umum	sexton		listrik		general workers pabrik		
	recruitment specialist	mandor	stock control	pelayan		elektrik		krew kapal		
	perencana	staff operasional	tukang pos	md		veneer		tenaga gudang		

1. Manager	2. Professionals	3. Technicians and associate professionals	4. Clerical support workers	5. Service and sales workers	6. Skilled agricultural, forestry and fishery workers	7. Craft and related trades workers	8. Plant and machine operators, and assemblers	9. Elementary occupations	10. Armed forces occupations	11. Other
	fisioterapi	invoice	laboratory information services	menchandiser		elektronika		tenaga kerja bangunan		
	orthoptist	pajak	customer service	merchandiser		tukang batu		abk kapal		
	instruktur	staff pajak	costumer service	merchandising		pandai logam		crew kapal		
	inspektur	r d staff	customer care	bartender		pandai besi		penjaga parkir		
	konselor	tax	customer servis	pemadam kebakaran		garber garments		kuli		
	tutor	pmo	customer relation officer	tele caller		gerber		ffice boy		
	educator	laboran	helpdesk	insurance agent		art work		bell boy		
	akuntan	maintainance	help desk	mitra usaha		reparasi sofa		bell service		
	pengembang	maentenance	costemer service	supervisor selling		tukang reparasi		door girl		
	development	maintanace	penerimaan service	staff konsultan		teknisi bengkel		docking kapal		
	developer	maintanace	tallyman	member relations officer		teknisi computer				
			penerimaan service							
	programer	support	pengelolaan data	mro		maintenance listrik				
	programmer	shipping	staf compliance	service attendant		supervisor amplas				
	administrator	eksim	field collection	customer contact		scrap staff				
	webmaster	procedur officer	collection	spa terapi		spv elektrikal				
	analis	personal assistant	collection officer	driving instructors		supervisor elektrik				
	analyst	personal assistan	media remainder sistem	pool attendants		spv elektro				
	analys	tenaga teknis	storage gudang	house keeping		tenaga sablon				
	spesialis	,	store inventory	guest service		bread baker				
	analisis data	marine repair	cinema crew	penjaga keamanan		baker				
	pustakawan	assistant agronomy	front office	staff promosi		pattern maker				
	sdm	quality control	payment point staff	hair stylist		supevisor produksi				
	librarian	qualinty control	operator telpon	penjaga pantai		supervisor produktion				
	arsiparis	quality controle	officer data service	account officer		supervisor production				
	kurator	quaity contrl	call center	tenaga penjual		spv produksi				
	penulis	quality assurance	hotline officer	tenaga market		tenaga jok				
	wartawan	assistant management	input data	credit officer		pembuat jok				
	journalist	koresponden	entri data	accoun officer		pembuat kursi				
	pengacara	proses control	petugas konter	trader		tenaga jahit				

1. Manager	2. Professionals	3. Technicians and associate professionals	4. Clerical support workers	5. Service and sales workers	6. Skilled agricultural, forestry and fishery workers	7. Craft and related trades workers	8. Plant and machine operators, and assemblers	9. Elementary occupations	10. Armed forces occupations	11. Other
	lawyer	bussiness control	team remedial	penyuluh lapangan		suppervisior cutting				
	hakim	product planing control	team tarik	petugas sentra		pembuat meubel				
	judge	material control	tata usaha	funding staff		sample maker				
	legal	desainer interior	pengantar pos	pembina sentra		supervisor listrik				
	jaksa	accountant staff	juru tik	ao		trainee electro				
	advokat	supervisor milling	record production	funding officer		mech plumbing				
	notaris	design interior	satff pdqc	kader		spv packing				
	psikoterapis	mantri kesehatan	exhebition team	server		spv for packaging				
	arkeolog	interior designer	assistant supervisor	merchant officer		packing supervisor				
	biarawan	pengawas kesehatan	pencatat meter	personal banker		tukang cat				
	biksu	rekam medik	dealing operational	dealer pulsa		tukang jahit				
	pendeta	servis area controller	credit control	pembina ekonomi mikro		spv amplas				
	imam	ass manager	pembantu umum	exspedisi boy		ahli pencicip				
	financial	ekspor impor	phone verifikator	pembina ekonomi mikro		spv jahit				
	penceramah	export import	supervisor bagian umum	valet service		personel avsec				
	editor	supplier office	follw up order	medical representavive		spv finising				
	penyair	hrga supervisor	room attendant	medical representative		supervisor jahit				
	novelist	hrd staff	casual concierge	medical represntativ		pengendali konstruksi				
	musisi	supervisor produksi	concierge	life assurance		flooring				
	copywriter	tenaga sipil	concierge casual	distributor pulsa		supervisor packaging				
	blogger	human resource assistant	stock control staff	medical representativ		supervisor moulding				
	reporter	production supervisor		medical represntative		supervisor carton box				
	presenter	spv produksi		supervisor creditcard		plumbing				
	interpreter	supervisor produsksi		supervisor credit card		fire fighting				
	kerohanian	maintenance supervisor		pengelola kantin		ducting				
	leksikograf	distribution logistic officer		account executive		supervisor ducting				
	ekonom	logistic officer		funding executive		chemical oil paints				

1. Manager	2. Professionals	3. Technicians and associate professionals	4. Clerical support workers	5. Service and sales workers	6. Skilled agricultural, forestry and fishery workers	7. Craft and related trades workers	8. Plant and machine operators, and assemblers	9. Elementary occupations	10. Armed forces occupations	11. Other
	ekonomi manajemen	spv logistik		quick service		elektrik weaving				
	subtitler	spv hospitality		bussines executive		tukang listrik				
	designer	maintenance		account receivabe officer		tenaga produksi				
	penerjemah	business improvement officer		account officers		staff listrik				
	translator	immunization officers		service officer		staff kontruksi				
	musisi	material planning		business executive		pekerja konstruksi				
	musician	perencanaan		business executives		insulation workers				
	penari	building supervisor		businees excecutif		welder				
	dancer	supervisor distribusi		telesales agent		tukang las				
	penyayi	construction supervisors		business relationship		karyawan amplas				
	doktor	supervisor operasional		nutritional promotor						
	pneumatics	supervisor teknik		direct selling						
	singer	asisten apoteker		staff ekspedisi						
	komposer	spv operational		partner dealer						
	composer	supervisor logistik		quick service staff						
	actor	supervisor rnd		fb service						
	aktor	hrga safety officer		building caretaker						
	announcer	supervisor pelayanan		team promo						
	penyiar	operation officer		pembina sentra						
	koreografer	safety officer		crew restaurant						
	kartunis	supervisor sipil		karyawan restoran						
	perancang	chinese cook		operasional resto						
	chef	koki		saff departement store						
	koki	juru masak		laundry attendant						
	pematung	edp staff		staff restaurant						
	orchestrator	supervisor listrik		restaurant crew						
	konduktor	supervisor pub		relationship anchor						
	sutradara	research staff		consultant						
	produser	product safety officer		nanny						

1. Manager	2. Professionals	3. Technicians and associate professionals	4. Clerical support workers	5. Service and sales workers	6. Skilled agricultural, forestry and fishery workers	7. Craft and related trades workers	8. Plant and machine operators, and assemblers	9. Elementary occupations	10. Armed forces occupations	11. Other
	pesulap	operasional		baby sitter						
	ventriloquist	tenaga pk		medical executive						
	dalang	rekrutmen		relationship officer						
	komedian	petty cash		bussiness consultant						
	aktuaria	staff safety		account funding officer						
	feldscher	training		konsultan marketing						
	ilmuwan	staff keuangan		personal investment assistant						
	journalistic	staff design		karyawan toko						
	cameraman	personal banking officer		pelayan restoran						
	marcom	supervisor chinesse restaurant		penjaga toko						
	marketing communication	supply chain management staff		card advisor						
	marketing supervisor	staf mis		alternate channel officer						
	public relation	staff ga		aco						
	external relations	tenaga perpajakan		jaga konter						
	cad drawing	staff general affair		karyawati toko						
	badut	tenaga riset lapangan		staff office						
	environtment analyst	supervisor store		terapi kesehatan						
	qc	cost control		building caretakers						
	desainer	supervisor r d		crew of store						
	engineer	laboratorium dyeing		financial consultant						
	enginnering	resourcing officer								
	engineering	staff akuntansi								
	enginering	staf keuangan								
	egineering	entry level								
	finance	i t staff								
	akunting	network assurance								
	akuntansi	relation officer								
	farmasi	costing								

1. Manager	2. Professionals	3. Technicians and associate professionals	4. Clerical support workers	5. Service and sales workers	6. Skilled agricultural, forestry and fishery workers	7. Craft and related trades workers	8. Plant and machine operators, and assemblers	9. Elementary occupations	10. Armed forces occupations	11. Other
	it	tenaga lapangan								
	i t	staff accounting								
	r d	spv logistik								
	market analisis	supervisor qa								
	pembelian	qa								
	purchasing	internal control								
		process engineer								
	network enginer	supervisor								
	bayer	supervisor marketing								
	purchase	supervisor teknis								
	personalia	project koordinator								
	hr	supervisor proyek								
	kacab	back office								
	safety specialist	mortgage officer								
	business development	spv logistik								
	aviation security	kordinator cabang								
	editing foto	supervisor lapangan								
	pr	pelaksana proyek								
	public affairs	staff humas								
	school counsellor	field supervisor								
	graphic design	supervisor outlet								
	desain graphic	supervisor toko								
	design grafis	supervisor spinning								
	desain grafis	staf gudang								
	design graphic	staff gudang								
	design grafhis	koordinator cabang								
	management transportasi	area control data								
	desain produk	koordinator lapangan								
	designer grafis	project control								
	tenaga pendidik	staff public relation								
	photo editing	laboratory information system								
	editor	supervisor card center								

1. Manager	2. Professionals	3. Technicians and associate professionals	4. Clerical support workers	5. Service and sales workers	6. Skilled agricultural, forestry and fishery workers	7. Craft and related trades workers	8. Plant and machine operators, and assemblers	9. Elementary occupations	10. Armed forces occupations	11. Other
	pelatih jahit	house keeping supervisor								
	technisi computer	supervisor sewing								
	furniture design	koordinasi umum								
	insinyur sipil	f b supervisor								
	market analist	data management supervisor								
	art work	warehouser								
	analisis kimia	knowledge management officer								
	pic plan concept	managemen officer								
	web designer	business officer								
	quality system	durty store supervisor								
	quality surveyor	store supervisor								
	park ranger	operasional supervisor								
	electrical engineering	koorwil								
	data analyt	exim staff								
	perancang busana	technikal supervisor								
	content writer	fix asset officer								
	biodiversity specialist	project assistant								
	pengembang multimedia	product assurance								
	analisis kesehatan	distribution logistic officer								
	disain grafis	management logistik								
	planning produktion	fo supervisor								
	industrial enginer	super intendent								
	pengkaji sosiologi	cinema supervisor								
	building inspections	supervisor penjualan								
	social specialist	engenering crew assistant								
	supply chain	packaging development manager								
	sistem informasi	advertising crew								

1. Manager	2. Professionals	3. Technicians and associate professionals	4. Clerical support workers	5. Service and sales workers	6. Skilled agricultural, forestry and fishery workers	7. Craft and related trades workers	8. Plant and machine operators, and assemblers	9. Elementary occupations	10. Armed forces occupations	11. Other
	management sistem sumber daya manusia<br>humas profesional<br>business consultant<br>product spesialist<br>clinical specialist<br>environment officer<br>general affair<br>visual artis<br>data processing<br>problem solving<br>industrial relation specialist<br>statistic analiyst<br>analisis lab<br>management information system<br>web design<br>web programming<br>teknik sipil<br>finsancial consultan<br>mesin analist<br>human resources officer<br>research team<br>praktisi medis<br>petugas medis<br>petugas klinis<br>consultant	project supervisor<br>site supervisor<br>transportation supervisor<br>product plaining control<br>petugas sterilisasi<br>brand acquistion<br>sourcing<br>sales supervisor<br>salaes supervisor<br>ka shift<br>sourcing raw material<br>engineering crew<br>ka regu<br>supervisor gudang<br>remedial officer<br>supervisor restaurant								
